# Furan Is Superior to Thiophene: A Furan‐Cored AIEgen with Remarkable Chromism and OLED Performance

**DOI:** 10.1002/advs.201700005

**Published:** 2017-02-27

**Authors:** Zheng Zhao, Han Nie, Congwu Ge, Yuanjing Cai, Yu Xiong, Ji Qi, Wenting Wu, Ryan T. K. Kwok, Xike Gao, Anjun Qin, Jacky W. Y. Lam, Ben Zhong Tang

**Affiliations:** ^1^ Department of Chemistry Hong Kong Branch of Chinese National Engineering Research Center for Tissue Restoration and Reconstruction Institute of Molecular Functional Materials State Key Laboratory of Nanoscience and Division of Biomedical Engineering The Hong Kong University of Science and Technology Clear Water Bay Kowloon Hong Kong China; ^2^ HKUST Shenzhen Research Institute Nanshan Shenzhen 518057 China; ^3^ State Key Laboratory of Luminescent Materials and Devices South China University of Technology Guangzhou 510640 China; ^4^ Key Laboratory of Synthetic and Self‐Assembly Chemistry for Organic Functional Molecules Shanghai Institute of Organic Chemistry Chinese Academy of Sciences 345 Lingling Road Shanghai 200032 China

**Keywords:** aggregation‐induced emission, chromism, diyne, furan, organic light‐emitting diodes

## Abstract

Furan‐cored AIEgen namely tetraphenylethylene‐furan (TPE‐F) is developed by diyne cyclization and its fluorescent and chemical properties are investigated and compared with its thiophene analogue. Results show that furan is superior to thiophene in terms of fluorescence, chromism, and charge transport. The mechanism of chromism of TPE‐F is investigated and its efficient solid‐state photoluminescence and good charge‐transporting property enable it to serve as light‐emitting material for the construction of electroluminescence devices with excellent performance. This work not only demonstrates an efficient strategy for constructing furan‐cored AIEgens but also indicates that they are promising as advanced optoelectronic materials.

## 1 Introduction

The past decade has witnessed a burgeoning development of organic semiconductors for applications in various advanced electronic devices such as organic field‐effect transistors (OFETs), organic photovoltaics (OPVs), and organic light‐emitting diodes (OLEDs), flexible displays, and sensors.^1^, ^2^, ^3^, ^4^, ^5^, ^6^, ^7^, ^8^ To achieve a fast progress of organic semiconductors, efficient organic π‐functional materials are required. Among them, those carrying thiophene moiety play a significant role.^9^, ^10^ In addition to synthetic flexibility, a common argument for the wide use of thiophene‐derived materials is the strong polarizability of the sulfur atoms which gives rise to strong S...S and S...π intermolecular interactions for contributing high charge mobility.^11^, ^12^ Based on this cognition, a large number of thiophene‐derived semiconductors were developed and were found to exhibit superior device performances in OFETs, OPVs, sensors, etc.

As an analogue of thiophene, furan and its derivatives are silent in organic electronics for years without attracting much attention. Furan was discovered in 1870 and was structural like thiophene. However, it exhibits chemical/physical properties quite different from those of thiophene.^13^ The higher electronegativity and smaller atomic size of oxygen relative to sulfur render furan less aromaticity and weak intermolecular heteroatom...heteroatom and heteroatom...π interactions. This leads to a misconception that furan‐based π‐functional materials are inferior than their thiophene counterparts.^13^, ^14^ However, recent studies demonstrated that furan derivatives could show comparable and even better charge‐transporting properties in addition to good solubility than their thiophene analogues.^15^, ^16^, ^17^, ^18^ It is speculated that the small atomic size of oxygen endows furan‐based π‐conjugates with smaller torsional angles and hence improved planarity, which is beneficial for molecular stacking in the solid state and charge transport.^19^, ^20^ More remarkably, furan derivatives do not suffer from the heavy atom effect observed in thiophene‐based luminogens. Thus, they tend to exhibit stronger luminescence and are promising candidates for fabricating advanced optoelectronic devices.^14^

Although furan‐based materials possess promising application in electronics, in comparison with the rich study history of thiophene, they are often overlooked by materials scientists.^13^, ^14^ In addition to the above misconception that furan is not as good as thiophene in terms of semiconducting property, the synthetic difficulty of its derivatives is also a big obstacle for its development. Oxidative coupling and metal‐mediated cross coupling are the main methodologies for the synthesis of furan‐containing π‐systems^13^ However, the first method is difficult to manipulate and easily lead to irreversible oxidative damage. On the other hand, metal‐mediated cross coupling such as Suzuki and Stille coupling often require the use of preactivated organometallic substrates (organic boron, organotin reagents, etc.) which are difficult to synthesize and toxic.^13^, ^14^, ^21^ As a result, exploration of new and efficient synthetic approaches or strategies for furan‐based π‐functional materials is highly desirable. Alkynes have been extensively utilized for constructing functional materials due to their rich, versatile, and efficient chemistry.^22^, ^23^ By cascaded nucleophilic addition of S^2−^ and OH^−^, respectively to diyne, thiophene, and furan conjugates were obtained in high yields.^24^, ^25^, ^26^, ^27^ While these reactions were developed for methodology study, they were scarcely utilized for material synthesis. Exploration of such possibility will open the door for the development of furan‐based π‐functional materials.

Traditional fluorophores usually suffer from the aggregation‐caused quenching (ACQ) effect: they are highly emissive in dilute solutions but emit no light at all when aggregated or in the solid state.^28^, ^29^ This greatly limits their practical applications since most of the devices function in the solid state. In 2001, Tang and his coworkers found a class of compounds with aggregation‐induced emission (AIE) characteristics, which showed negligible emission in dilute solutions but enhanced fluorescence in the aggregated state due to the restriction of intramolecular motion (RIM).^28^, ^29^, ^30^ The discovery of AIE has elegantly solved the ACQ problem of traditional fluorophores. Guided by the RIM mechanism, many AIE luminogens (AIEgens) with different emissions and various applications have been designed and synthesized. However, few furan‐cored AIEgens have been prepared although they may possess both the advantages of strong emission and good charge‐transporting properties.^30^ We have recently investigated the emission of tetraphenyl‐substituted furan (TPF) and compared it with its thiophene analogue (TPT).^31^ We found that TPF showed a much stronger fluorescence than TPT in tetrahydrofuran (THF), which was ascribed to its higher conjugation. TPF was not emissive in the solid state although it showed a propeller structures similar to silole and tetraphenylethylene (TPE). These results suggest that the incorporation of furan into the π‐conjugated system endows the resulting compound with improved emission in the solution state but is challenging to produce an AIEgen.

In this contribution, we presented the synthesis of furan‐containing luminogen (TPE‐F) with AIE characteristic and chromic property by cycloaddition of OH^−^ to diyne. The synthesis could be further simplified by a one‐pot reaction using terminal alkyne as substrate. For comparison, thiophene‐containing AIEgen (TPE‐T) was also synthesized by the same method. The photophysical properties and mechanism of the chromism of TPE‐F were investigated in details by studying its radiative and nonradiative processes in the excited state and crystallographic analysis. Comparison with its thiophene analog was also made. Results indicated that furan was superior to thiophene in constructing high‐performance fluorescent and chromic materials. The efficient solid‐state photoluminescence (PL) and better charge‐transporting property of TPE‐F enabled it to serve as an excellent light‐emitting material for the construction of electroluminescence (EL) devices. OLED device utilizing TPE‐F as the light‐emitting layer showed excellent device performance with high luminance of 24 298 cd m^−2^ and EL efficiencies of 9.98 cd A^−1^ and 3.67%, which was among the best for heterocyclic‐bridged TPE derivatives.

## 2 Results and Discussion

**Scheme 1:**
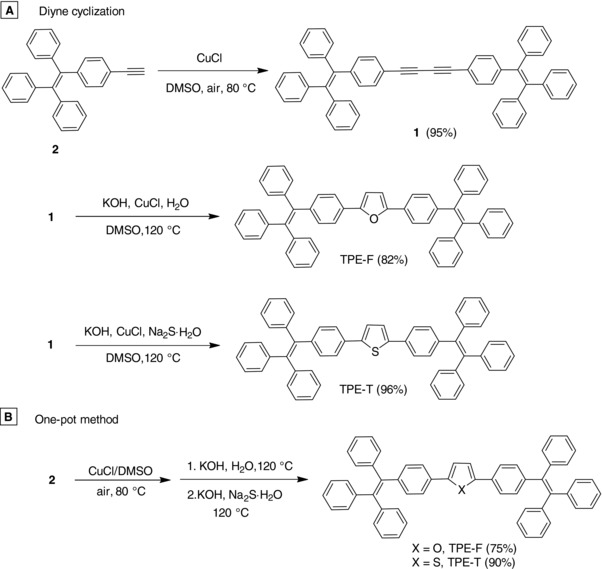
Synthetic routes for TPE‐F and TPE‐T.

The synthetic strategy for TPE‐F and TPE‐T is shown in **Scheme** **1**. Compound **1** was prepared by Glaser coupling of compound **2**. Compound **1** then underwent cycloaddition in the presence of nucleophilic agent (S^2−^ or OH^−^) and CuCl as catalyst, affording TPE‐F and TPE‐T in high yields. Since the two reaction steps proceeded with the same catalyst and solvent, one‐pot reaction was further implemented which gave rise to the same target compounds in satisfactory yields (Scheme 1B). Both TPE‐F and TPE‐T were carefully purified and fully characterized by NMR and mass spectroscopies (Figures S1−S6 in the Supporting Information). Their single crystals were obtained by slow evaporation of their dichloromethane (DCM)/hexane mixtures at ambient conditions and were analyzed crystallographically. TPE‐F and TPE‐T dissolved readily in common organic solvents (such as DCM, chloroform, and tetrahydrofuran) and showed high thermal stability, losing merely 5% of their weight at high temperature of 388 and 407 °C, respectively (Figure S7, Supporting Information). The glass‐transition temperature of TPE‐F and TPE‐T determined by differential scanning calorimetry was found at 129 and 125 °C, respectively (Figure S2, Supporting Information), which was suggestive of their high morphological stability (Figure S8, Supporting Information).

**Figure 1:**
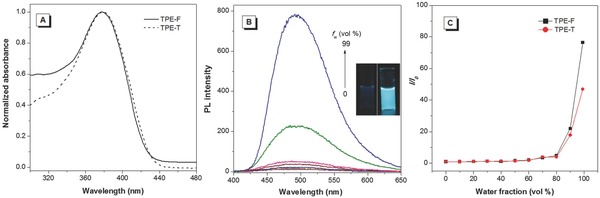
A) UV–vis absorption spectra of TPE‐F and TPE‐T in dilute THF solutions (10 × 10^−6^ m). B) PL spectra of TPE‐F (10 × 10^−6^ m) in THF/water mixtures with different water fractions (*f*_w_). C) Plot of relative emission intensity (*I*/*I*_0_) versus composition of the THF/water mixtures of TPE‐F and TPE‐T. *I*_0_ = PL intensity in pure THF.

The optical properties of TPE‐F and TPE‐T were characterized by UV–vis and PL spectroscopies. The UV spectra of TPE‐F and TPE‐T were similar and were peaked at 378 nm, which was indicative of their similar electronic structure in the ground state. Thanks to the introduction of TPE, both TPE‐F and TPE‐T showed typical AIE characteristic: their weak emission in dilute THF solutions was enhanced upon aggregate formation. The AIE characteristic of TPE‐F and TPE‐T were further demonstrated by studying their PL behaviors in THF and THF/water mixtures with different water fractions (*f*_w_). In dilute THF solution, both TPE‐F and TPE‐T were weakly emissive owing to the active intramolecular motion, which consumed the energy of the excited state through nonradiative relaxation pathway (**Figure** **1**B,C; Figure S9, Supporting Information). Addition of a large amount of water into their THF solutions aggregated their molecules and enhanced their PL. The highest emission was achieved at a *f*_w_ of 99%. Compared with TPE‐T, the aggregates of TPE‐F showed a stronger PL, possibly due to the greater restriction in the intramolecular motion by a more compact molecular packing. The quantum yields of TPE‐F and TPE‐T in dilute THF solutions were determined to be 3.5% and 3.7% by a calibrated integrating sphere. In the solid state, their quantum yields were measured to be 50% and 18%. These results indicate that TPE‐F shows a more efficient emission than TPE‐T in the solid state, demonstrating that furan is more favorable in affording luminophores with stronger emission than thiophene. The optical data and thermal properties of TPE‐F and TPE‐T are summarized in **Table** **1**.

**Table 1:** Optical and thermal properties of TPE‐F and TPE‐T. λ_em_ = emission maximum; *Φ*_F_ = fluorescence quantum yield; HOMO = highest occupied molecular orbitals; LUMO = lowest unoccupied molecular orbitals; *T*_d_ = temperature for 5% weight loss measured by TGA; *T*_g_ = glass transition temperature determined by DSC; λ_abs_ = absorption maximum

		λ_em_ [nm]		*Φ*_F_ [%]				
	λ_abs_ [nm] Soln.	Soln.	Aggregate	Soln.	Solid	HOMO [eV]	LUMO [eV]	*T*_d_/*T*_g_ [°C]
TPE‐F	378	488	491	3.5	50	−5.31	−2.41	388/129
TPE‐T	378	479	487	3.7	18	−5.41	−2.51	407/125

To better understand the AIE characteristics of TPE‐F and TPE‐T and stronger fluorescence of TPE‐F, we measured their fluorescence lifetime in solution and solid state and investigated the solid‐state radiative and nonradiative decay processes (Figure S10, Supporting Information; **Table** **2**). From the solution to the solid state, the radiative decay rate (*k*_r_) of TPE‐F increased by around two times (*k*_r,soln_ = 0.79 × 10^8^ s^−1^ and *k*_r,solid_ = 1.4 × 10^8^ s^−1^), while the nonradiative decay rate (*k*_nr_) decreased almost by 16 times (*k*_nr,soln_ = 21.9 × 10^8^ s^−1^ and *k*_nr,solid_ = 1.4 × 10^8^ s^−1^). This suggests that the inhibition of nonradiative decay of excited state in the solid state is accounted for its high fluorescence. In sharp contrast, the *k*_r_ of TPE‐T increased by around 2.6 times upon aggregate formation, while its *k*_nr_ decreased by only about 2.3 times, whose extent was much smaller than that in TPE‐F. Evidently, the incorporation of furan can effectively restrict the nonradiative decay of the excited state in a greater extent than thiophene does, generating AIEgens with stronger fluorescence.

**Table 2:** Fluorescence lifetime and decay of TPE‐F and TPE‐T. 〈τ〉 = fluorescence lifetime; *k*_nr_ = nonradiative decay rate; *k*_r_ = radiative decay rate

	〈τ〉 [ns]		*k*_r_/*k*_nr_ [×10^8^ s^−1^]	*k*_r_/*k*_nr_ [×10^8^ s^−1^]
	Soln.	Solid	Soln.	Solid
TPE‐F	0.44	3.57	0.79/21.9	1.4/1.4
TPE‐T	0.50	0.96	0.74/19.3	1.9/8.5

**Figure 2:**
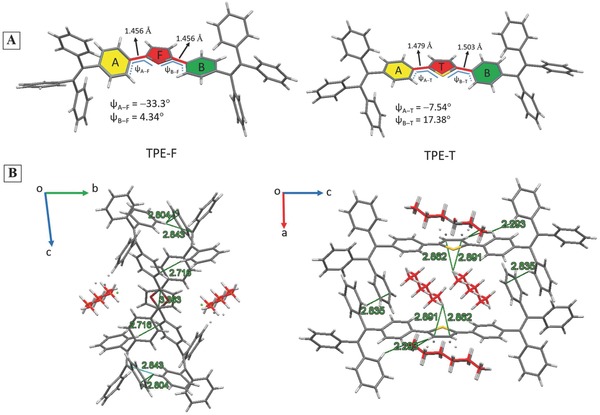
A) Single‐crystal structures and B) intermolecular interactions in the crystal lattice of TPE‐F and TPE‐F.

The single‐crystal structure of TPE‐F and TPE‐T and the associated crystallographic data are given in **Figure** **2** and Figure S11, Tables S1 and S2 (Supporting Information). The torsion angles (ψ) between furan and the adjacent phenyl rings in TPE‐F (ψ_A−F_ = −33.30 and ψ_B−F_ = 4.34) and those in TPE‐T (ψ_A−T_ = −7.54 and ψ_B−T_ = 14.38) are suitable for conjugation.^32^ Interestingly, the bond lengths between furan and the adjacent phenyl rings in TPE‐F (1.456 and 1.456 Å) were shorter than those in TPE‐T (1.479 and 1.503Å), indicative of better conjugation in the former molecule. Gidron and Bendikov demonstrated that oligofurans showed good conjugation due to the high rigidity and less aromaticity of furan.^14^ These two factors may make the double bonds of the furan ring more available for electronic communication, leading to better conjugation in TPE‐F in our case.^13^, ^14^ The solvent molecules of hexane and DCM were preserved in the crystals of both TPE‐F and TPE‐T. In TPE‐F crystal, abundant intermolecular interactions such as C—H...C=C (distance: 2.843 Å), C—H...π (distance: 2.804 Å), π...π (distance: 3.363 Å), etc., were found, which helped rigidify the molecular conformation and made TPE‐F strongly emissive in the crystal state. In TPE‐T, only some C—H...solvent interactions (2.862, 2.891, 2.293 Å) and few C—H...π interaction (distance: 2.835 Å) were found, which revealed a loosely packed structure. Closer examination indicated that the average volume occupied by a single molecule in the crystal lattice of TPE‐F (2126.99 Å^3^) was much smaller than TPE‐T (4996.7 Å^3^). The molecular density (ρ_calc_) of the unit cell was calculated to be ρ_calc_ = 1.215 g cm^−3^ for TPE‐F and ρ_calc_ = 1.162 g cm^−3^ for TPE‐T. Thus, the larger PL increase in aggregates of TPE‐F than TPE‐T could be ascribed to the denser packing of TPE‐F, which restricted the intermolecular motions in a greater extent.

**Figure 3:**
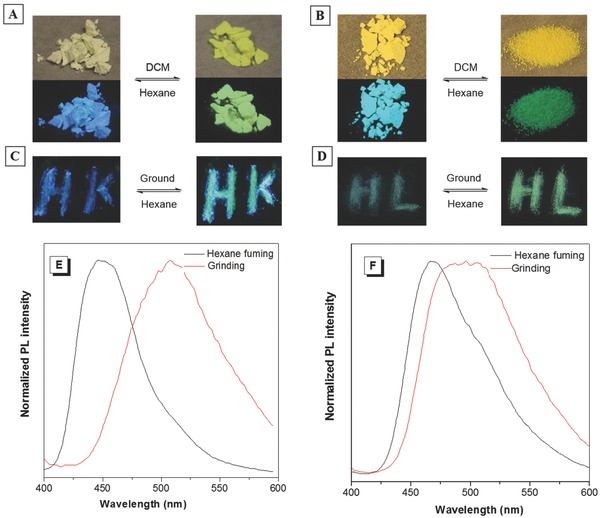
Switching the color and solid‐state emission of A,C) TPE‐F and B,D) TPE‐T by solvent fuming and grinding processes. E,F) PL spectra change of TPE‐F and TPE‐T by solvent fuming and grinding processes.

Chromic materials are a class of smart materials which alter their absorption or emission by changing their molecular conformation, morphology, or crystallinity in the presence of external stimuli. They have attracted great attention for their importance in both fundamental research and practical applications.^33^, ^34^, ^35^ Many AIE‐active materials have been reported with reversible mechanochromic or thermochromic properties, but the associated mechanism for guiding their design and synthesis has not been well investigated.^34^ We thus studied the chromic properties of TPE‐F and TPE‐T and explained the change in their chromic properties in the presence of external perturbation by X‐ray diffraction and crystallographic analysis. Both TPE‐F and TPE‐T exhibited color and fluorescence changes upon solvent fuming and mechanical grinding due to the interconversion between the crystalline and amorphous phases upon external perturbation and vice versa. Compared with TPE‐T, TPE‐F showed larger color and fluorescence contrast (**Figure** **3**; Figure S12, Supporting Information). For example, the crystalline powder of TPE‐F precipitated from hexane was white in color and showed deep blue luminescence. After fuming with DCM or mechanical grinding, the powder was amorphorized, redshifting its color and fluorescence to yellow and yellowish‐green, respectively. The powder of TPE‐T turned to pale yellow color and emitted sky‐blue light after fumed by hexane, while that treated with DCM vapor fuming or mechanical grinding force appeared yellow and now showed green fluorescence. By measuring their PL spectra after treated with solvent vapor, we found that the emission of TPE‐F redshifted by 61 nm, while TPE‐T showed a PL redshift of 30 nm. The longer‐wavelength absorption and emission indicate that the molecular structure becomes more planar when transformed from crystalline to amorphous state. A greater conformational change may occur in TPE‐F when an external stimulus is applied, thus explaining its bigger chromic contrast than TPE‐T. It is also worthy to note that the fluorescence of ground powder of TPE‐F can be fully recovered to the original blue one in the absence of hexane vapor fuming, as supported by the recuperative diffraction peaks appeared in its X‐ray diffractogram after 12 h storage. This may be stemmed by the multi‐intermolecular interactions and denser molecular packing of TPE‐F in the solid state, which are beneficial to induce molecular conformational adjustment and rearrangement to the crystalline state.^36^ It is worthy to note that the PL spectrum of TPE‐F in the solid state is different from that in aggregate suspension, which can be explained by their different aggregation extent and crystallinity.

**Figure 4:**
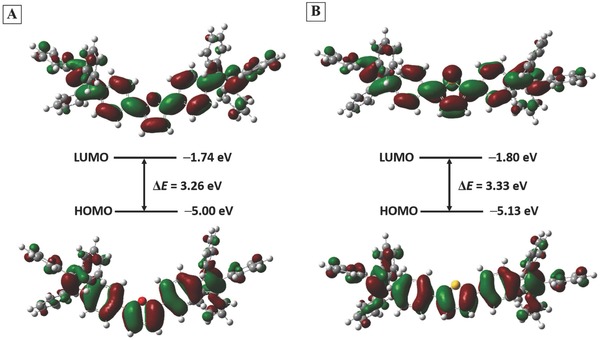
HOMO and LUMO orbital distributions, energy level, and band gaps of A) TPE‐F and B) TPE‐T obtained by DFT calculations.

To gain more insight into the electronic structures of TPE‐F and TPE‐T, we carried out molecular simulation at the DFT B3LYP/6‐31G* level with the Gaussian 09 package. In both TPE‐F and TPE‐T, the largest coefficients in the highest occupied molecular orbitals (HOMO) were located along the central π‐backbones (**Figure** **4**). This indicates that furan can also work as excellent bridge to enhance the electronic communication between segments as thiophene. The HOMO and lowest unoccupied molecular orbitals (LUMO) of TPE‐F and TPE‐T were well overlapped, thus contributing their strong emission. The HOMO and LUMO of TPE‐F estimated by DFT calculation were −1.74 and −5.00 eV, which were comparable to those of TPE‐T (HOMO = −1.80 and LUMO = −5.13). This suggests their similar electronic structures. The HOMO and LUMO of the molecules were also determined by cyclic voltammetry (CV) measurement (Figure S13, Supporting Information). Both TPE‐F and TPE‐T showed two reversible or quasireversible oxidation waves with onset oxidative potentials of 0.83 and 1.03 V, respectively. The HOMO/LUMO of TPE‐F and TPE‐T estimated from CV were −5.31/−2.41 and −5.41/−2.51 eV, respectively. The higher HOMO energy level of TPE‐F is an advantage for hole injection. The charge‐transporting properties of TPE‐F and TPE‐T were evaluated from their OFET devices with top‐gate bottom‐contact configurations (Figure S14, Supporting Information) and the corresponding data were summarized in Table S3 (Supporting Information). The luminogens films were deposited by spin‐coating method of their DCM solutions, which gave an amorphous film. The hole mobility of TPE‐F was two orders magnitude higher than TPE‐T, thanks to its better charge injection and more compact molecular packing.

**Figure 5:**
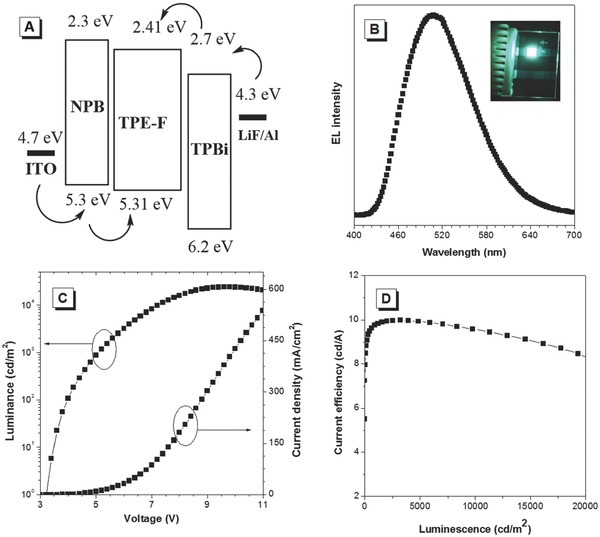
A) EL device configuration of TPE‐F: ITO/NPB/TPE‐F/TPBi/LiF/Al. B) EL spectrum. C) Current density–voltage–luminance characteristics. D) Change in external quantum efficiency with the applied current density in multilayer EL devices of TPE‐F. Inset: photo of the device.

The strong solid‐state PL, higher charge transporting property and good thermal and morphological stabilities of TPE‐F inspire us to investigate its EL properties. A nondoped multilayer EL device with a configuration of indium tin oxide (ITO)/N,N′‐Di(1‐naphthyl)‐N,N′‐diphenyl‐(1,1′‐biphenyl)‐4,4′‐diamine (NBP) (60 nm)/TPE‐F (20 nm)/TPBi (40 nm)/LiF (1 nm)/Al (100 nm) (device I) was fabricated by vapor deposition process, in which TPE‐F served as emitter, NPB worked as hole‐transporting layer, 2,2′,2″‐(1,3,5‐benzinetriyl)tris(1‐phenyl‐1‐H‐benzimidazole) (TPBi) functioned as both a hole‐blocking and electron‐transporting material, respectively. **Figure** **5** shows the EL spectrum and the device performance. The device radiated bright green light with a low turn‐on voltage of 3.3 V. The maximum luminescence (*L*), current efficiency (η_c_), power efficiency (η_P_), and external quantum efficiency attained by the device were 24 298 cd m^−2^, 9.98 cd A^−1^, 7.02 lm W^−1^, and 3.67%, respectively. Compared those AIEgens bridged with other heteroaromatics such as benzo‐2,1,3‐thiadiazole and silole with the same device structure, the performance of TPE‐F was better.^37^, ^38^ This implies that furan cored‐AIEgens are promising candidates for advanced optoelectronic materials.

## 3 Conclusion

In summary, a furan‐cored AIEgen with bright emission, remarkable chromism, and favorable charge‐transporting property was synthesized by cascaded cyclization of diyne. The photophysical properties and mechanism of chromism of TPE‐F were investigated in details and compared with its thiophene analogue. Results showed that the extent of molecular planarity and intermolecular interactions as well as the packing density were responsible for giving different aggregates with varied color and emission. Based on its efficient solid‐state PL, charge transportation, and high thermal and morphological stabilities, nondoped OLED device of TPE‐F was fabricated which exhibited low turn‐on voltage, high brightness, and outstanding EL efficiencies, demonstrating the great potential of furan‐cored AIEgens in advanced optoelectronics.

## 4 Experimental Section

*Synthesis of TPE‐F and TPE‐T by Diyne Cyclization*: To a round bottom flask were added compound **2** (100 mg, 0.14 mmol), CuCl (0.7 mg, 5% mmol), and KOH (2 mg, 0.04 mmol) in dimethylsulfoxide (DMSO) (2 mL), and H_2_O (0.1 mL) or Na_2_S⋅9H_2_O (33.6 mg, 0.14 mmol). The mixture was stirred at 120 °C for 30 min, giving TPE‐F in a yield of 82% or TPE‐T in quantitative yield.

*Synthesis of TPE‐F and TPE‐T by One‐Pot Method*: A mixture of compound **2** (200 mg, 0.56 mmol) and CuCl (3 mg, 5% mmol) in DMSO (4 mL) was stirred at 80 °C for 8 h, follow which KOH (31 mg, 0.56 mmol) and H_2_O (0.2 mL) or KOH (9.4 mg, 0.17 mmol) and Na_2_S⋅9H_2_O (33.6 mg, 0.14 mmol) were added. After stirring at 120 °C for 30 min, TPE‐F and TPE‐T were obtained in 75% and 90% yields, respectively. TPE‐F and TPE‐T were purified by column chromatography and characterized by standard spectroscopic methods.

TPE‐F: ^1^H NMR (400 MHz, CDCl_3_, δ): 7.45−7.43 (d, *J* = 8 Hz, 4H), 7.20−7.09 (m, 36H); ^13^C NMR (100 MHz, CDCl_3_, δ): 153.3, 143.7, 143.6, 142.9, 141.2, 131.8, 131.4, 131.3, 128.7, 127.8, 127.7, 127.6, 126.5, 126.4, 122.9, 107.3; HRMS (MALDI‐TOF): *m*/*z*: [M]^+^ calcd for C_56_H_40_O, 728.3079; found, 728.3107.

TPE‐T: ^1^H NMR (400 MHz, CDCl_3_, δ): 7.43−7.40 (d, *J* = 8 Hz, 4H), 7.11−7.02 (m, 34H), 6.62 (s, 2H); ^13^C NMR (100 MHz, CDCl_3_, δ): 143.7, 143.6, 143.2, 143.0, 141.2, 140.3, 132.2, 131.9, 131.4, 131.3, 127.8, 127.7, 127.6, 126.6, 126.5, 126.4, 124.7, 123.8; HRMS (MALDI‐TOF): *m*/*z*: [M]^+^ calcd for C_56_H_40_S, 744.2851; found, 744.2873.

[CCDC 1519798 (TPE‐F) and 1519799 (TPE‐T) contains the supplementary crystallographic data for this paper. These data can be obtained free of charge from The Cambridge Crystallographic Data Centre via www.ccdc.cam.ac.uk/data_request/cif.]

## Supporting information

Supplementary

## References

[1] L. Lu , T. Zheng , Q. Wu , A. M. Schneider , D. Zhao , L. Yu , Chem. Rev. 2015, 115, 12666. 26252903 10.1021/acs.chemrev.5b00098

[2] Y. Tao , K. Yuan , T. Chen , P. Xu , H. Li , R. Chen , C. Zheng , L. Zhang , W. Huang , Adv. Mater. 2014, 26, 7931. 25230116 10.1002/adma.201402532

[3] V. Jankus , M. Aydemir , F. B. Dias , A. P. Monkman , Adv. Sci. 2016, 3, 1500221. 10.1002/advs.201500221 PMC4991292 27610333

[4] X. Gao , Z. Zhao , Sci. China Chem. 2015, 58, 947.

[5] G. Schwartz , B. C. K. Tee , J. Mei , A. L. Appleton , D. H. Kim , H. Wang , Z. Bao , Nat. Commun. 2013, 4, 1859. 23673644 10.1038/ncomms2832

[6] Y. Chu , X. Wu , J. Lu , D. Liu , J. Du , G. Zhang , J. Huang , Adv. Sci. 2016, 3, 1500435. 10.1002/advs.201500435 PMC5069582 27812481

[7] B. Wang , T.‐P. Huynh , W. Wu , N. Hayek , T. T. Do , J. C. Cancilla , J. S. Torrecilla , M. M. Nahid , J. M. Colwell , O. M. Gazit , S. R. Puniredd , C. R. McNeill , P. Sonar , H. Haick , Adv. Mater. 2016, 28, 4012. 26996398 10.1002/adma.201505641

[8] X. Wang , L. Dong , H. Zhang , R. Yu , C. Pan , Z. L. Wang , Adv. Sci. 2015, 2, 1500169. 10.1002/advs.201500169 PMC5115318 27980911

[9] M. E. Cinar , T. Ozturk , Chem. Rev. 2015, 115, 3036. 25831021 10.1021/cr500271a

[10] J. Mei , Y. Diao , A. L. Appleton , L. Fang , Z. Bao , J. Am. Chem. Soc. 2013, 135, 6724. 23557391 10.1021/ja400881n

[11] G. R. Hutchison , M. A. Ratner , T. J. Marks , J. Am. Chem. Soc. 2005, 127, 16866. 16316233 10.1021/ja0533996

[12] O. Gidron , A. Dadvand , E. Wei‐Hsin Sun , I. Chung , L. J. W. Shimon , M. Bendikov , D. F. Perepichka , J. Mater. Chem. C 2013, 1, 4358.

[13] B. C. Streifel , J. D. Tovar , Encyclopedia of Polymer Science and Technology, John Wiley & Sons, Inc, New York, USA, 2012.

[14] O. Gidron , M. Bendikov , Angew. Chem., Int. Ed. 2014, 53, 2546. 10.1002/anie.201308216 24470351

[15] P. Huang , J. Du , M. C. Biewer , M. C. Stefan , J. Mater. Chem. A. 2015, 3, 6244.

[16] Y. Xiong , J. Tao , R. Wang , X. Qiao , X. Yang , D. Wang , H. Wu , H. Li , Adv. Mater. 2016, 28, 5949. 27167524 10.1002/adma.201600120

[17] P. Sonar , S. P. Singh , E. L. Williams , Y. Li , M. S. Soh , A. Dodabalapur , J. Mater. Chem. 2012, 22, 4425.

[18] C. H. Woo , P. M. Beaujuge , T. W. Holcombe , O. P. Lee , J. M. J. Fréchet , J. Am. Chem. Soc. 2010, 132, 15547. 20945901 10.1021/ja108115y

[19] O. Gidron , A. Dadvand , Y. Sheynin , M. Bendikov , D. F. Perepichka , Chem. Commun. 2011, 47, 1976. 10.1039/c0cc04699j 21165466

[20] X.‐H. Jin , D. Sheberla , L. J. W. Shimon , M. Bendikov , J. Am. Chem. Soc. 2014, 136, 2592. 24437464 10.1021/ja411842g

[21] A. Hucke , M. P. Cava , J. Org. Chem. 1998, 63, 7413. 11672391 10.1021/jo981159l

[22] J. Liu , J. W. Y. Lam , B. Z. Tang , Chem. Rev. 2009, 109, 5799. 19678641 10.1021/cr900149d

[23] B. Z. Tang , Macromol. Chem. Phys. 2008, 209, 1303.

[24] J. Tang , X. Zhao , RSC Adv. 2012, 2, 5488.

[25] G. Zhang , H. Yi , H. Chen , C. Bian , C. Liu , A. Lei , Org. Lett. 2014, 16, 6156. 25409534 10.1021/ol503015b

[26] I. Talbi , C. Alayrac , J.‐F. Lohier , S. Touil , B. Witulski , Org. Lett. 2016, 18, 2656. 27184563 10.1021/acs.orglett.6b01101

[27] Q. Zheng , R. Hua , J. Jiang , L. Zhang , Tetrahedron 2014, 70, 8252.

[28] J. Yang , L. Li , Y. Yu , Z. Ren , Q. Peng , S. Ye , Q. Li , Z. Li , Mater. Chem. Front. 2017, 1, 91.

[29] B. S. Li , R. Wen , S. Xue , L. Shi , Z. Tang , Z. Wang , B. Z. Tang , Mater. Chem. Front. 2017, DOI:10.1039/C6QM00120C.

[30] J. Mei , N. L. C. Leung , R. T. K. Kwok , J. W. Y. Lam , B. Z. Tang , Chem. Rev. 2015, 115, 1718. 10.1021/acs.chemrev.5b00263 26492387

[31] H. Nie , K. Hu , Y. Cai , Q. Peng , Z. Zhao , R. R. Hu , J. Chen , S. Su , A. Qin , B. Z. Tang , Mater. Chem. Front. 2017, DOI: 10.1039/C6QM00343E.

[32] S. Sharma , M. Bendikov , Chem. Eur. J. 2013, 19, 13127. 23940018 10.1002/chem.201300257

[33] Y. Q. Dong , J. W. Y. Lam , B. Z. Tang , J. Phys. Chem. Lett. 2015, 6, 3429. 26268912 10.1021/acs.jpclett.5b01090

[34] Z. He , L. Zhang , J. Mei , T. Zhang , J. W. Y. Lam , Z. Shuai , Y. Q. Dong , B. Z. Tang , Chem. Mater. 2015, 27, 6601.

[35] C. Ge , Y. Liu , X. Ye , X. Zheng , Q. Han , J. Liu , X. Tao , Mater. Chem. Front. 2017, DOI: 10.1039/c6qm00146g.

[36] Y. Lin , G. Chen , L. Zhao , W. Z. Yuan , Y. Zhang , B. Z. Tang , J. Mater. Chem. C 2015, 3, 112.

[37] Z. Zhao , C. Deng , S. Chen , J. W. Y. Lam , W. Qin , P. Lu , Z. Wang , H. S. Kwok , Y. Ma , H. Qiu , B. Z. Tang , Chem. Commun. 2011, 47, 8847. 10.1039/c1cc12775f 21709905

[38] Z. Zhao , S. Chen , J. W. Y. Lam , C. K. W. Jim , C. Y. K. Chan , Z. Wang , P. Lu , C. Deng , H. S. Kwok , Y. Ma , B. Z. Tang , J. Phys. Chem. C 2010, 114, 7963.

