# Supplementary material for: Furan Is Superior to Thiophene: A Furan‐Cored AIEgen with Remarkable Chromism and OLED Performance
Source: Adv Sci (Weinh). 2017 Feb 27;4(8):1700005. doi: 10.1002/advs.201700005 (PMC5566239; doi:10.1002/advs.201700005)
Supplement: Supplementary file 1 — Supplementary [file ADVS-4-na-s001.pdf]

Copyright WILEY-VCH Verlag GmbH & Co. KGaA, 69469 Weinheim, Germany, 2017.

ADVANCED SCIENCE
Open Access

Supporting Information

for *Adv. Sci.,* DOI: 10.1002/advs.201700005

Furan Is Superior to Thiophene: A Furan-Cored AIEgen with Remarkable Chromism and OLED Performance

*Zheng Zhao, Han Nie, Congwu Ge, Yuanjing Cai, Yu Xiong, Ji Qi, Wenting Wu, Ryan T. K. Kwok, Xike Gao, Anjun Qin, Jacky W. Y. Lam, and Ben Zhong Tang**

((Supporting Information can be included here using this template))

Copyright WILEY-VCH Verlag GmbH & Co. KGaA, 69469 Weinheim, Germany, 2016.

Supporting Information

Furan Is Superior to Thiophene: A Furan-cored AIEgen with Remarkable Chromism and OLED Performance

*Zheng Zhao, Han Nie, Congwu Ge, Yuanjing Cai, Yu Xiong, Ji Qi, Wenting Wu, Ryan T. K. Kwok, Xike Gao, Anjun Qin, Jacky W. Y. Lam and Ben Zhong Tang**

Dr Z. Zhao, Dr J. Qi, Dr R. T. K. Kwok, Dr J. W. Y. Lam, Prof. B. Z. Tang
Department of Chemistry, Hong Kong Branch of Chinese National Engineering Research Center for Tissue Restoration and Reconstruction, Institute of Molecular Functional Materials, State Key Laboratory of Nanoscience and Division of Biomedical Engineering, The Hong Kong University of Science and Technology, Clear Water Bay, Kowloon, Hong Kong, China.
E-mail: tangbenz@ust.hk
H. Nie, Dr Y. Cai, Prof. A. Qin, Prof. B. Z. Tang
State Key Laboratory of Luminescent Materials and Devices, South China University of Technology, Guangzhou 510640, China.
C. Ge, W. Wu, Prof. X. Gao
Key Laboratory of Synthetic and Self-Assembly Chemistry for Organic Functional Molecules, Shanghai Institute of Organic Chemistry, Chinese Academy of Sciences, 345 Lingling Road, Shanghai 200032, China.
Dr Z. Zhao, Dr Y. Xiong, Prof. B. Z. Tang
HKUST Shenzhen Research Institute Nanshan, Shenzhen 518057 , China

Keywords: aggregation-induced emission, furan, chromism, diyne, organic light-emitting diodes

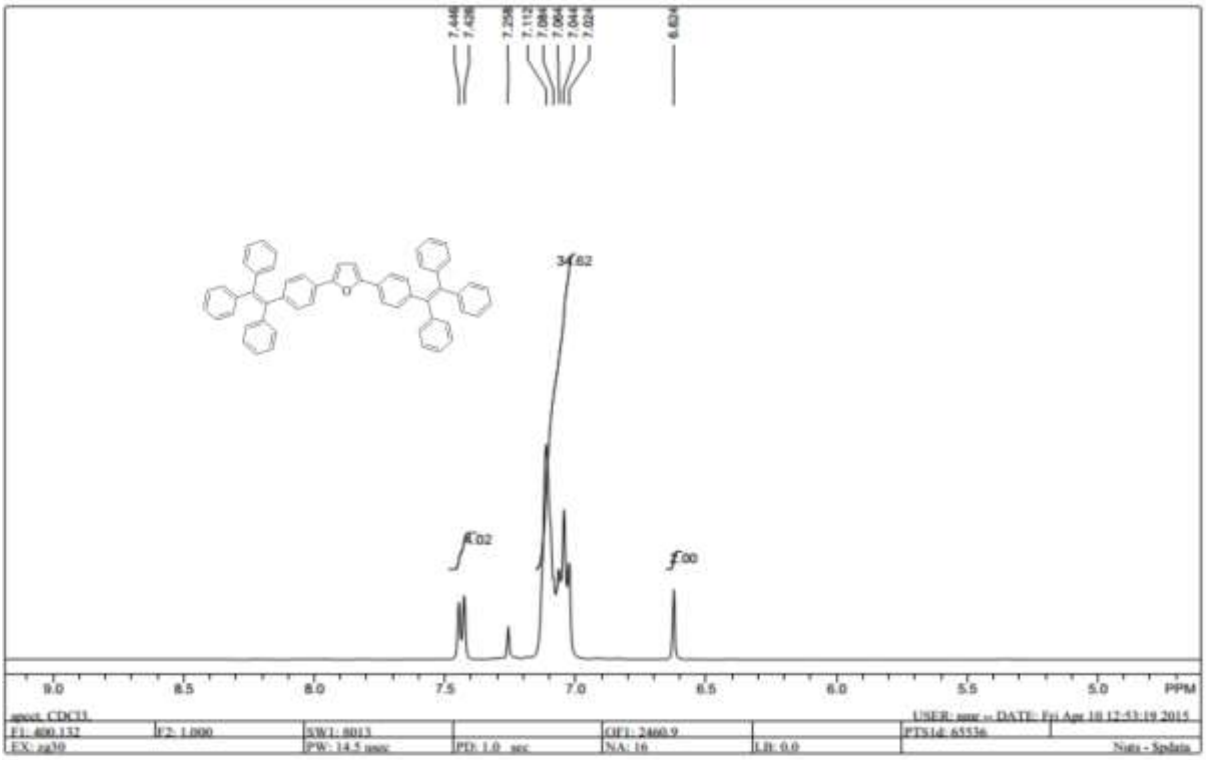

Figure S1. ^{1}H NMR spectrum of TPE-F in $CDCl_3$.

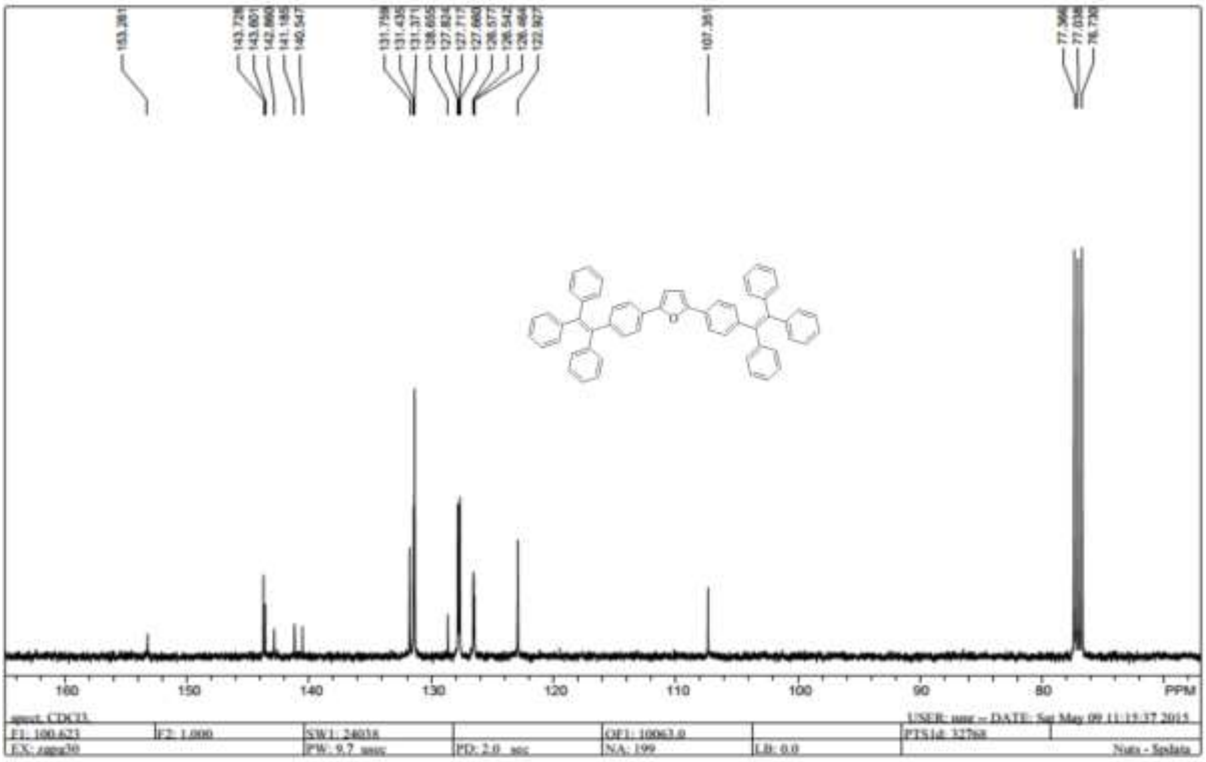

Figure S2. ^{13}C NMR spectrum of TPE-F in $CDCl_3$.

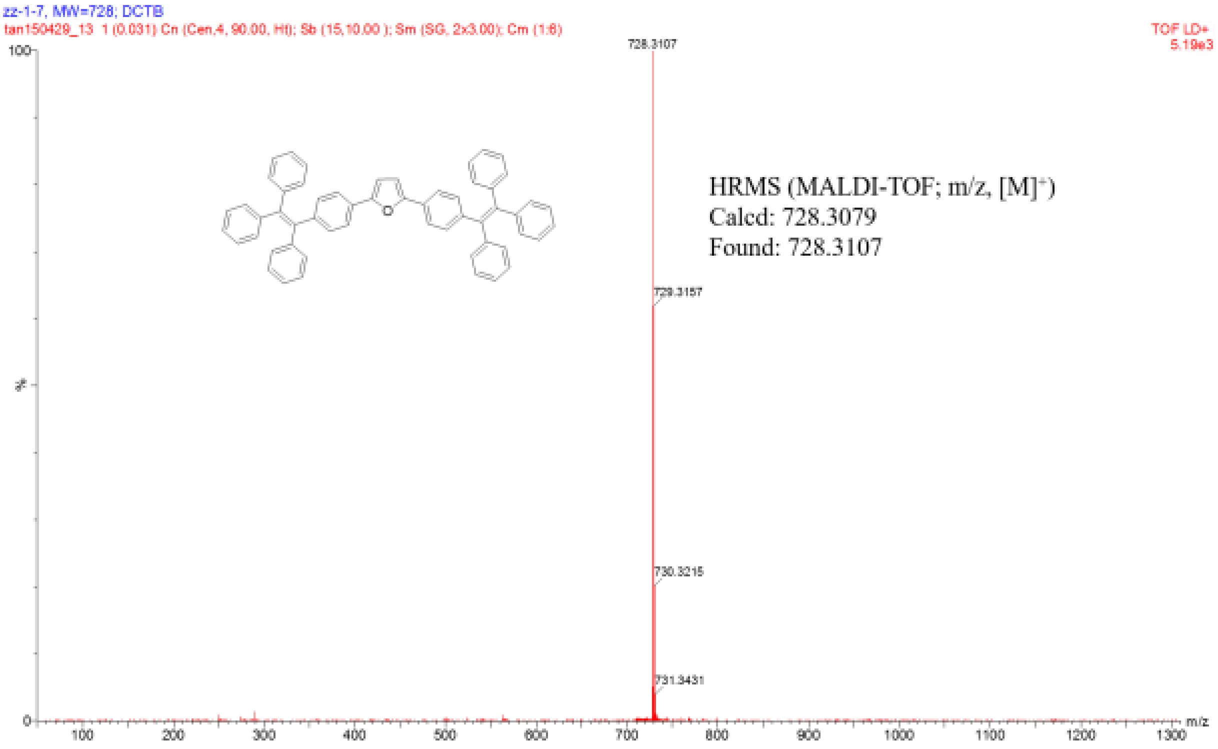

Figure S3. High resolution mass spectrum of TPE-F.

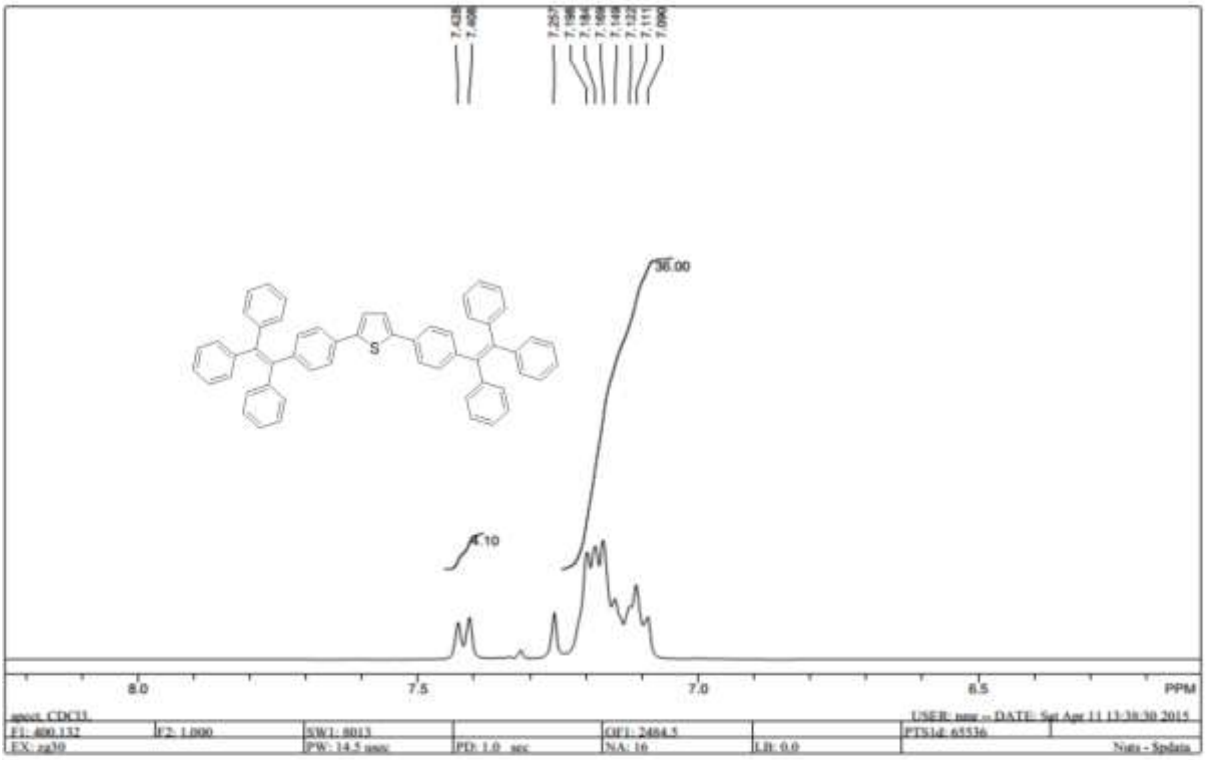

Figure S4. ^{1}H NMR spectrum of TPE-T in $CDCl_3$.

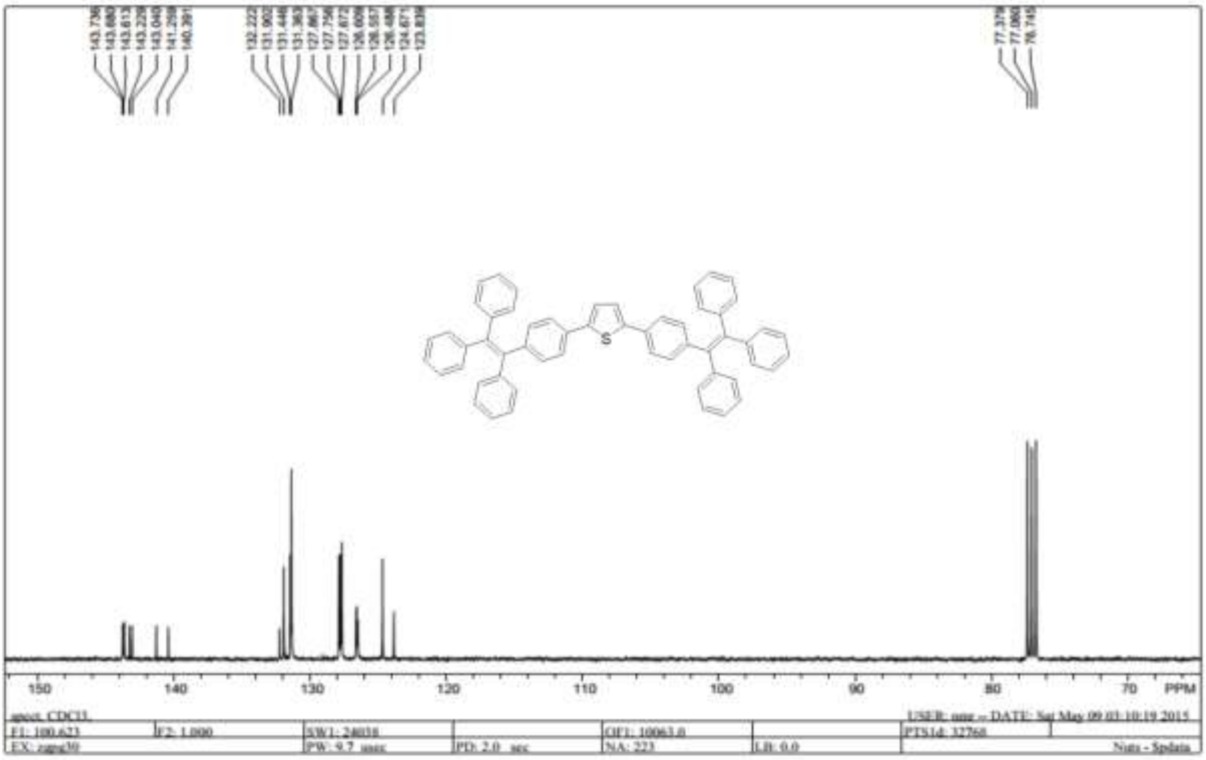

Figure S5. ^{13}C NMR spectrum of TPE-T in $CDCl_3$.

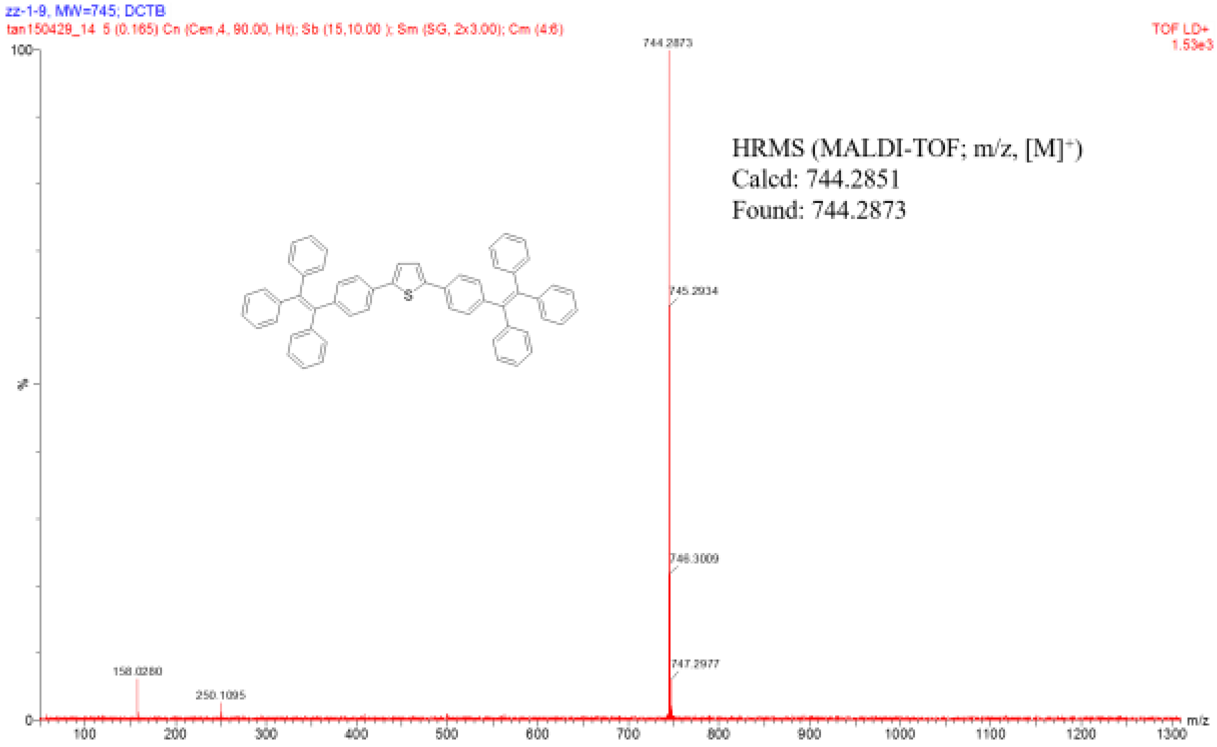

Figure S6. High resolution mass spectrum of TPE-T.

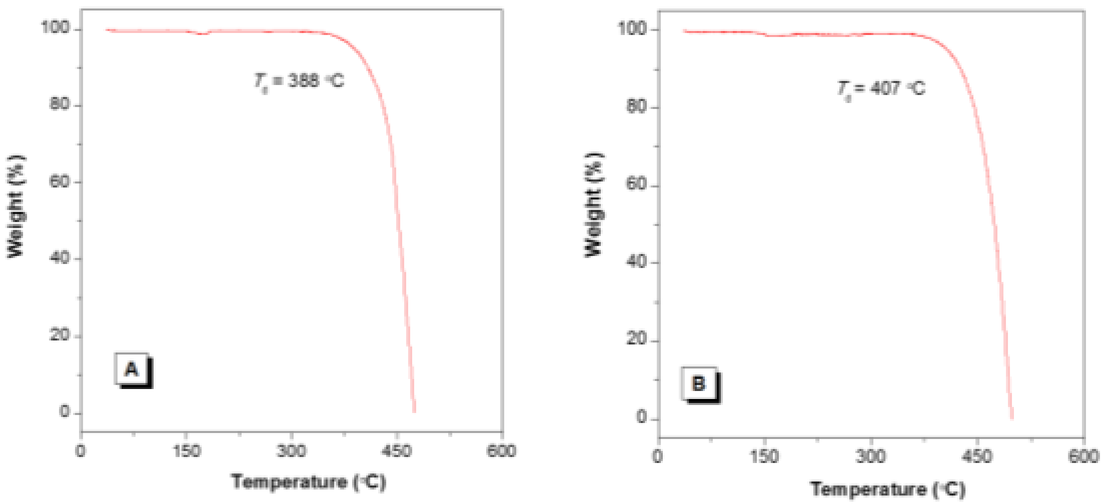

Figure S7. TGA curves of (A) TPE-F and (B) TPE-T recorded under nitrogen at a heating rate of 10 °C/min.

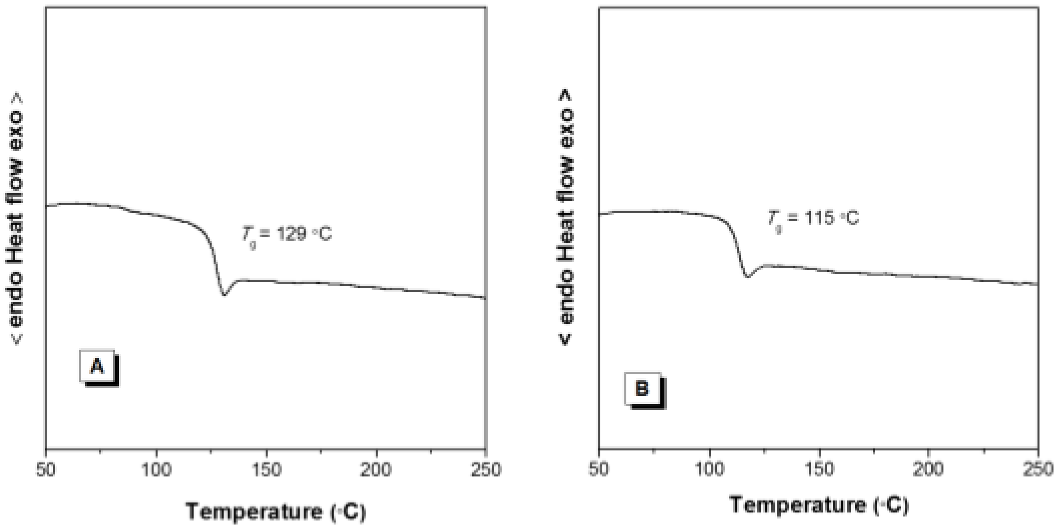

Figure S8. DSC thermograms of (A) TPE-F and (B) TPE-T recorded during the second heating cycle under nitrogen at a heating rate of 10 °C/min.

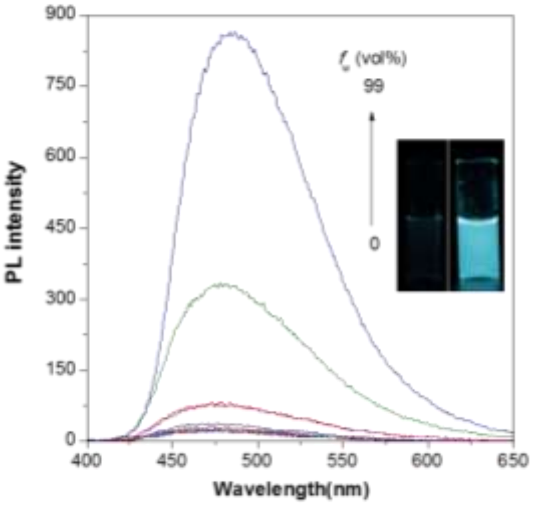

Figure S9. PL spectra of TPE-T (10 μM) in THF/water mixtures with different water fractions (f_w). Inset: fluorescence photos of TPE−T in dilute THF solution and at 99% aqueous mixture taken under 365 nm UV irradiation from a hand-hold lamp.

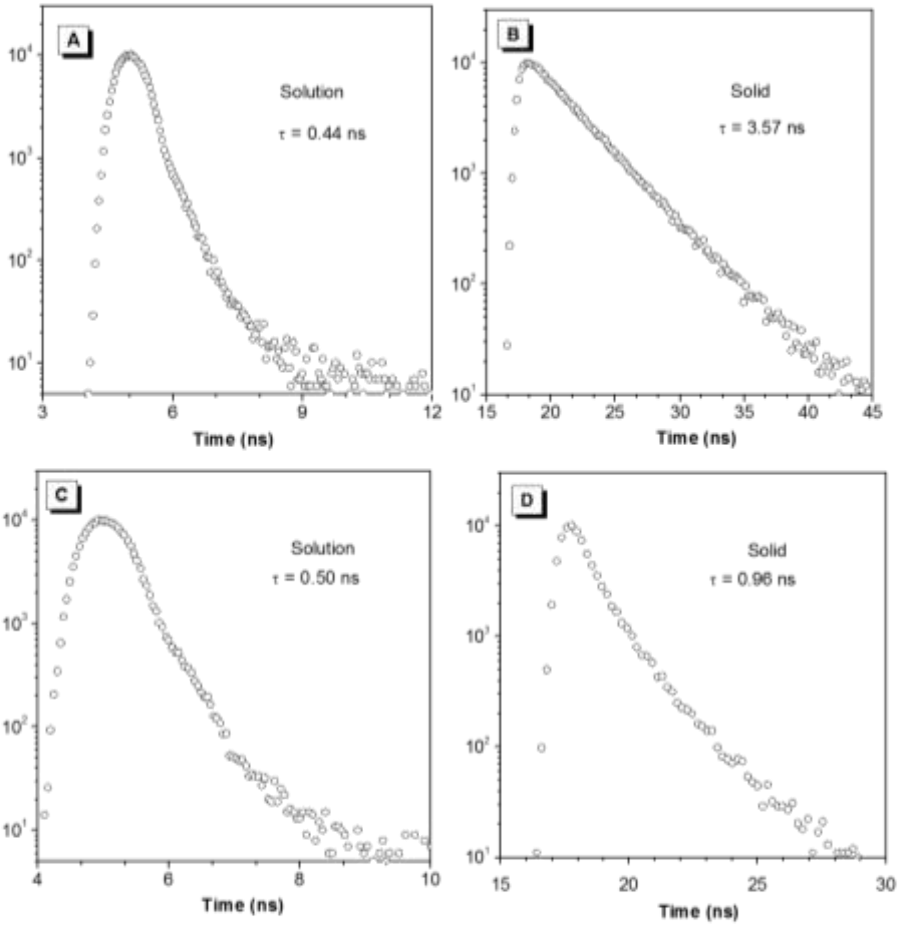

Figure S10. Fluorescence decay curves of (A and B) TPE-F and (C and D) TPE-T in the solution and solid states.

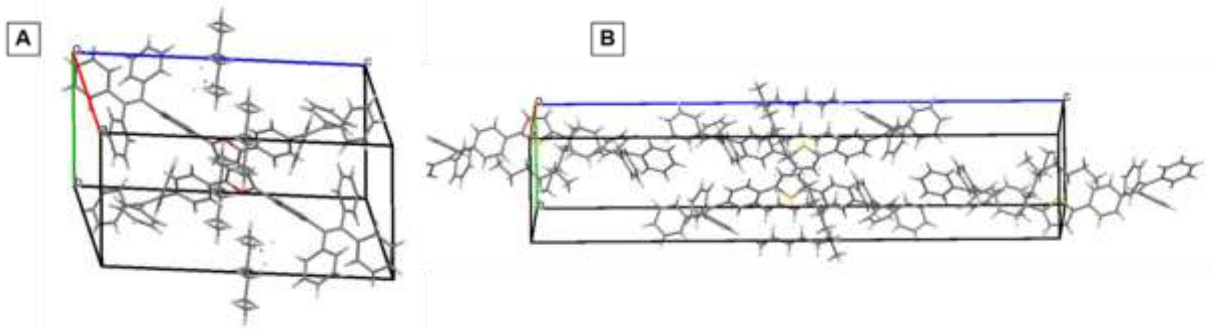

Figure S11. Unit cells of (A) TPE-F and (B) TPE-T and their cell parameters.

Table S1. Crystal data and structure refinement for TPE-F.

Identification code	TPE-F
Empirical formula	$C_{58.7}H_{46.25}Cl_{0.3}O$
Formula weight	778.24
Temperature/K	99.94(16)
Crystal system	triclinic
Space group	P-1
a/Å	9.2650(4)
b/Å	11.3333(5)
c/Å	20.5731(8)
α/°	82.002(4)
β/°	84.209(3)
γ/°	87.183(4)
Volume/Å^3	2126.96(16)
Z	2
ρ_{calc}g/cm^3	1.215
μ/mm^{-1}	0.704
F(000)	823.0
Crystal size/mm^3	0.25 × 0.2 × 0.2
Radiation	CuKα (λ = 1.54184)
2Θ range for data collection/°	8.478 to 134.966
Index ranges	-11 ≤ h ≤ 10, -13 ≤ k ≤ 13, -18 ≤ l ≤ 24
Reflections collected	12047
Independent reflections	7558 [R_{int} = 0.0169, R_{sigma} = 0.0266]
Data/restraints/parameters	7558/2/554
Completeness to theta = 66.5°	98.9%
Goodness-of-fit on F^2	1.000
Final R indexes [I>=2σ (I)]	R_1 = 0.0397, wR_2 = 0.0968
Final R indexes [all data]	R_1 = 0.0462, wR_2 = 0.1011
Largest diff. peak/hole / e Å^{-3}	0.34/-0.43

Table S2. Crystal data and structure refinement forTPE-T.

Identification code	TPE-T
Empirical formula	$C_{65}H_{61}S$
Formula weight	874.19
Temperature/K	100.01(10)
Crystal system	monoclinic
Space group	$P2_1/n$
a/Å	12.0793(3)
b/Å	9.1542(2)
c/Å	45.1926(10)
α/°	90
β/°	90.888(2)
γ/°	90
Volume/Å^3	4996.7(2)
Z	4
ρ_{calc}g/cm^3	1.162
μ/mm^{-1}	0.869
F(000)	1868.0
Crystal size/mm^3	0.2 × 0.2 × 0.08
Radiation	CuKα (λ = 1.54184)
2Θ range for data collection/°	9.314 to 133.996
Index ranges	$-14 \le h \le 14$, $-8 \le k \le 10$, $-53 \le l \le 50$
Reflections collected	26520
Independent reflections	8799 [R_{int} = 0.0317, R_{sigma} = 0.0296]
Data/restraints/parameters	8799/30/603
Completeness to theta = 66.5°	99.0%
Goodness-of-fit on F^2	1.001
Final R indexes [I>=2σ (I)]	R_1 = 0.0778, wR_2 = 0.1922
Final R indexes [all data]	R_1 = 0.0866, wR_2 = 0.1984
Largest diff. peak/hole / e Å^{-3}	0.51/-0.50

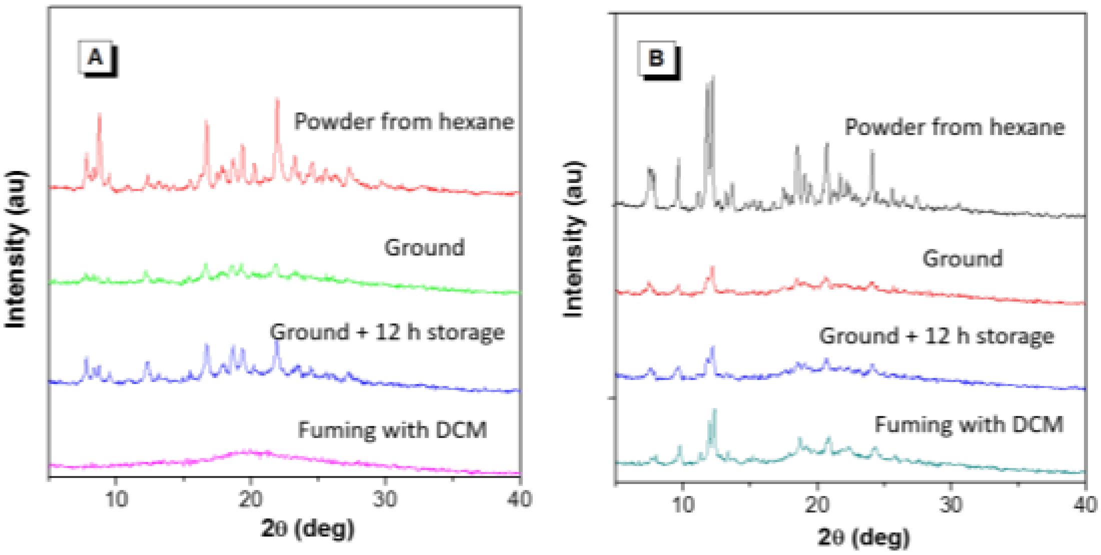

Figure S12. (A) Powder XRD diffractiongrams of (A) TPE-F and (B) TPE-T before and after grinding, DCM fuming and stroage after 12 h.

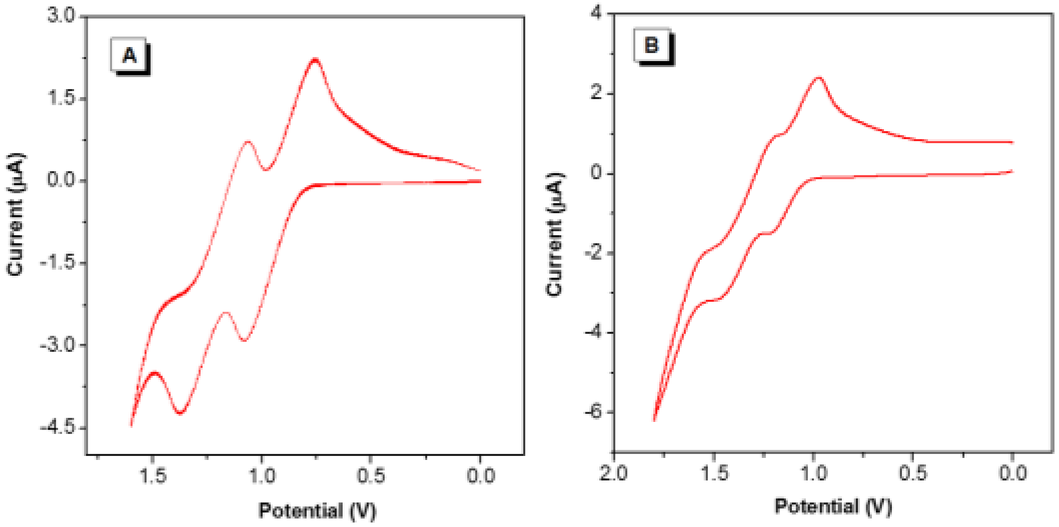

Figure S13. Cyclic voltammetry curves of (A) TPE-Fand (B) TPE-T with 0.1 M $Bu_4^+NPF_6^-$ in CH_2Cl_2 solution at a scan rate of 100 mV/s.

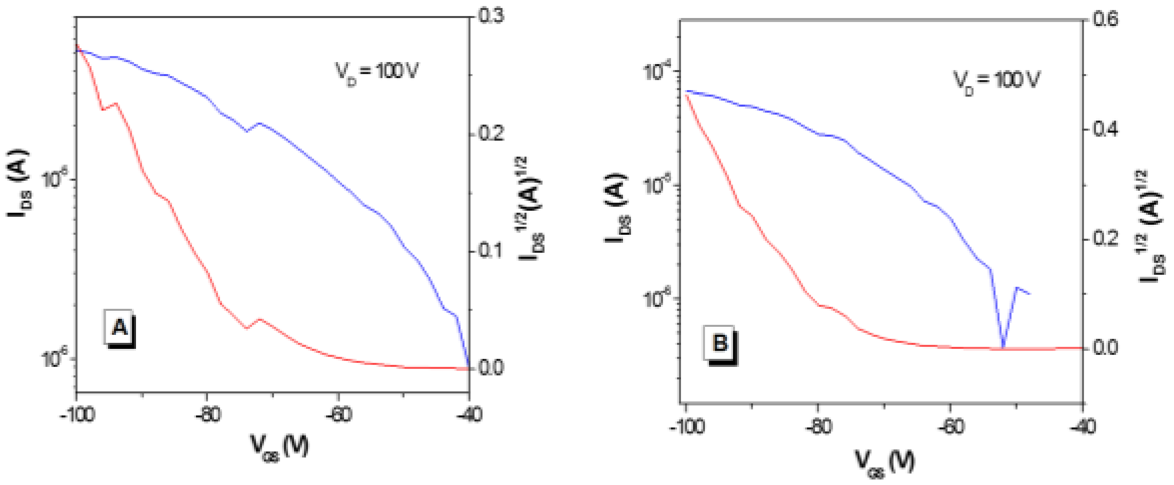

Figure S14. Transfer characteristics of organic field-effect transistor based on (A) TPE-F and (B) TPE-T.

Table S3. Characteristics of OFETs based on TPE-F at different annealing temperatures.

Active layer	T (°C)	V_T (V)	I_{on}/I_{off}	μ_h (max) (10^{-4} $cm^2/V{\cdot}s$)
TPE-F	25	−36	10^4	0.35
	80	−37	10^4	0.7
	120	−37	10^3	0.37
TPE-T	25	−51	10^2	0.0075
	80	−61	10^3	0.0043
	120	−37		
